# Large-Scale MOCVD Deposition of Nanostructured TiO_2_ on Stainless Steel Woven: A Systematic Investigation of Photoactivity as a Function of Film Thickness

**DOI:** 10.3390/nano12060992

**Published:** 2022-03-17

**Authors:** Alessandro Galenda, Marta Maria Natile, Naida El Habra

**Affiliations:** 1 ICMATE-CNR Institute of Condensed Matter Chemistry and Technologies for Energy, National Research Council, Corso Stati Uniti, 4, 35127 Padova, Italy; naida.elhabra@cnr.it; 2 ICMATE-CNR Institute of Condensed Matter Chemistry and Technologies for Energy, National Research Council, Department of Chemical Science, University of Padova, Via F. Marzolo, 1, 35131 Padova, Italy; martamaria.natile@cnr.it

**Keywords:** TiO_2_ film thickness, MOCVD, large area substrate, photocatalysis, ISO 10678:2010

## Abstract

Heterogeneous photocatalysis is considered as one of the most appealing options for the treatment of organic pollutants in water. However, its definitive translation into industrial practice is still very limited because of both the complexity of large-scale production of catalysts and the problems involved in handling the powder-based photocatalysts in the industrial plants. Here, we demonstrate that the MOCVD approach can be successfully used to prepare large-scale supported catalysts with a good photocatalytic activity towards dye degradation. The photocatalyst consisted of nanostructured TiO_2_ thin film deposited on a stainless steel mesh substrate. The film thickness, the morphological features, and the crystallographic properties of the different portions of the sample were correlated to the position in the reactor chamber and the reaction conditions. The photocatalytic activity was evaluated according to the international standard test ISO 10678:2010 based on methylene blue degradation. The photocatalytic activity is essentially constant (P_MB_ over 40 µmol·m^−2^·h^−1^) throughout the film, except for the portion of sample placed at the very end of the reactor chamber, where the TiO_2_ film is too thin to react properly. It was assessed that a minimum film thickness of 250–300 nm is necessary to reach the maximum photocatalytic performance.

## 1. Introduction

Heterogeneous photocatalyst for wastewater treatment in a lab setting is commonly studied as a suspended powder. This procedure requires a further processing step to remove the catalyst from aqueous suspensions and recover it for reuse. In an attempt to circumvent these costs and favour the implementation in industrial practice, a catalyst immobilization consisting in the coating of a suitable substrate with a layer of photocatalyst has been proposed [1,2]. The use of large supported (photo)catalysts, in fact, could overcome the limitations of nano- and micro-powders employment.

The photocatalytic properties of TiO_2_ have been well recognized since long time. In particular, the application of TiO_2_ thin film in photocatalysis for water and air remediation have been well-known since the late 1960s and is well-reviewed [3,4,5,6].

The deposition of TiO_2_ thin film by low pressure metal-organic chemical vapour deposition (LP-MOCVD) has been widely investigated; nevertheless, dedicated literature seems to be lacking when the up-scale of the sample size is taken into account. The evaluation of the photocatalytic properties of the laboratory-scale samples (usually a few square centimetres) is the first step for addressing and optimizing the synthetic efforts, of course, but they do not guarantee the same performance on a large-area sample. As of the publication date, no relevant records are found in Web of Science when “TiO_2_ and MOCVD and large sample” or “TiO_2_ and MOCVD and upscale” or “TiO_2_ and MOCVD and large substrate” strings are used for search. Only one record is found using “industrial scale up and TiO_2_ and MOCVD” as key words [7]. Moreover, the papers are mainly focused on atmospheric pressure or pulsed pressure reactors [7,8,9] rather than LP-MOCVD. Atmospheric pressure reactors are more attractive from an industrial perspective because of their simplified management. Nevertheless, the homogeneity of the deposition is usually lower than the one obtained with low pressure reactors. A film deposition on larger substrates is not straightforward. It needs accurate planning to ensure a high film homogeneity, a crucial feature for the specific properties required for the desired application. Usually, a gradual “step-increase” of the sample size can give more useful information than the direct optimization of the final target sample size. Moreover, other strategies can be helpful, such as working on reactors’ geometries, possible sample movement during the film growth, precursor stream (single-stream, multi-streams, and precursor amount), temperature management, etc. Thompson et al. [10] investigated the enlargement in substrate dimension for rotating disk reactors for AlGaAs and InGaAlP layers up to 420 mm disk diameter. Yang et al. [11] proposed a novel super-large MOCVD reactor for GaN deposition, while Meysami et al. [12] optimised a large area (90 cm^2^) aerosol assisted CVD for multi-wall carbon nanotube on silicon wafer. 

In this scenario, the novelty of this work lies in facing the upscaling of the samples for approaching the need of an actual water treatment plant in pilot scale.

The TiO_2_ deposition on large area stainless steel mesh is investigated and the photocatalytic properties of the obtained system are assessed. As stated above, indeed, the simple widening of the sample and reactor dimensions is not enough for replacing the small-area sample properties. Because of this, rectangular stainless steel mesh samples with 320 cm^2^ geometrical area (40 × 8 cm^2^) were functionalized with TiO_2_ thin film by LP-MOCVD, and the photocatalytic activity was explored as a function of the growing position with respect to the ISO 10678:2010 protocol [13]. Thanks to this systematic approach, it is possible to evaluate and mapping the photocatalytic performance of significant portions of the wide-area sample, thus establishing the most active and the most critical zones. The accurate correlation between the photocatalytic activity and the sample properties (obtained from its characterization) allows the determinations of the key factors affecting photocatalytic performances.

Although ISO 10678:2010 employs methylene blue as probe molecule (instead of more updated or interesting probes), it was chosen as a sharable, reproducible, and inter-laboratory comparable methodology which appears very useful for a preliminary evaluation of the photocatalytic performance of the prepared samples. A stainless steel mesh was selected as substrate in view of its subsequent employment in photo-reactors for water or air treatment for pollutants removal. The morphology and the thickness of the film were characterized by means of scanning electron microscopy (FE-SEM), while the structural properties were determined by X-ray diffraction (XRD) to identify the optimal TiO_2_ film properties ensuring suitable photocatalytic activity.

## 2. Materials and Methods

### 2.1. Deposition of Nanostructured TiO_2_ on Stainless Steel Mesh

Stainless steel mesh (AISI 316) was purchased from a local provider (Gaudenzi srl, Albignasego, Padova, Italy). The bare mesh (named as SSM) was a weave made of crossed wires with a diameter of 30 µm and the distance between the wires was 30 µm. These geometrical characteristics lead to about 2.9 cm^2^ of total effective area each square centimetre of weave (the overlapping zones were subtracted). Before the TiO_2_ deposition, several 6 to 8 cm wide rectangular substrates with different lengths (maximum 50 cm) were cut from the stainless-steel mesh. Each portion of bare mesh was washed with water and soap, then rinsed with deionised water and dried at room temperature in air.

Titanium tetra-isopropoxide [Ti(O^i^Pr)_4_] (TTIP, 97%, with O^i^Pr = -CH(CH_3_)_2_) from Sigma-Aldrich was used as the precursor (without any further purification). MOCVD experiments were carried out in a horizontal large area hot-wall reactor. The reactor oven had a cylindrical hot-zone 70 cm of total length and 10 cm of diameter. The oven was equipped with three independent heating zones (each one about 1/3 of the total length), controlled by three K-type thermocouples. The reactor temperatures were set at 350, 380 and 400 °C starting from the reactor inlet to optimise the precursor consumption (and thus the film growth homogeneity) along the deposition chamber. The titanium precursor tank was heated at 50 °C as the ideal value for an optimal TTIP supply in deposition chamber, without any over-supply and carried throughout the reactor by a 100 sccm nitrogen flow. The pipeline between precursor tank and reactor was kept at 65 °C in order to avoid condensation along the line before entering in the reactor zone. The total pressure during deposition was 65 Pa and the deposition time was 1500 s for obtaining a substantial film thickness in the widest region of the sample surface. The obtained samples were named as TiO_2_-SSM.

### 2.2. Characterizations

The TiO_2_-SSM mesh, as well as the bare SSM mesh, were characterised by different analytical techniques. The structural characterisation was determined via X-ray Diffraction (XRD) performed by means of a (Bruker D8 Advance Plus, Bruker, Karlsruhe, Germany), diffractometer operating in a Bragg-Brentano θ-θ geometry, using Cu Kα radiation λ = 1.5406 Å, 40 kV and 40 mA. Standard 2002 ICDD database files supported the phase identification. The crystallite dimensions were estimated by Scherrer’s equation calculated with respect to the main reflections (101) and (200) of TiO_2_ in anatase phase. The surface morphology of the samples was investigated by FEI Quanta 200 FEG-ESEM scanning electron microscopy (SEM, FEI, Hillsboro, OR, USA), supplied with a field emission gun, operating in high vacuum conditions and at an accelerating voltage of 20 kV.

### 2.3. Photocatalytic Measurements

The photocatalytic properties of the selected mesh portions have been analysed with respect to the ISO standard 10678:2010 [13] for methylene blue removal, as also described in a previous work [14]. Briefly, the ISO 10,678 regulates the determination of photocatalytic activity of surfaces in aqueous medium by degradation of methylene blue. The results are given, as required by ISO standard, in terms of specific photoactivity P_MB_ (µmol·m^−2^·h^−1^), calculated by the equations provided in the protocol. The standard imposes the use of UVA lamp in the range 320–400 nm (Philips PL-S 9W/2P BLB at 370 nm in the present case, Philips, Amsterdam, Holland) with 10 ± 0.5 W/m^2^ measured at the height of the sample. The power density of the lamps was measured by a Delta Ohm HD 2302.0 light-meter equipped with a LP471PA probe. The measurements were performed at 22 °C, in a beaker filled with 50 mL MB solution (10 μmol/L for test solution and 20 μmol/L for conditioning solution; pH = 5.5−6.0, i.e., the natural pH of MB solution in distilled water); sample surface = 4.20 cm^2^. The measurements were carried out following the standard protocol, as well as the data collection and processing (photocatalytic test was no longer than 3 h), while the MB solution absorbance was measured using a Shimadzu UV-2600 spectrophotometer (Shimadzu, Kyoto, Japan).

## 3. Results and Discussion

### 3.1. SSM Substrate and TiO_2_-SSM Samples Characterisation

**Figure 1:**
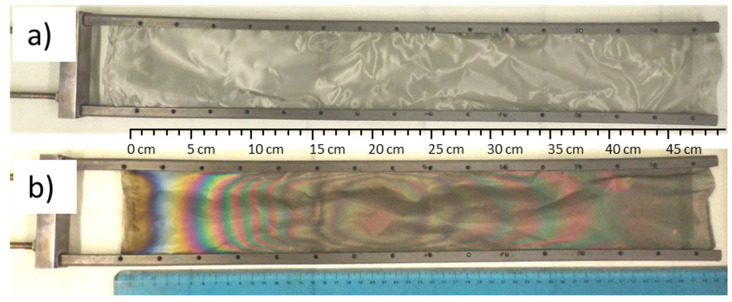
SSM bare substrate (**a**) and TiO_2_-SSM (**b**) fixed to the MOCVD sample holder.

The bare SSM and the TiO_2_-SSM samples were characterised by different points of view. Figure 1 shows the SSM before (a) and after (b) the TiO_2_ deposition.

Figure 1b helps in analysing the TiO_2_ film characteristics. The single-source TTIP/N_2_ feed enters into the MOCVD reactor from the right side, then reacts and settles as TiO_2_ on the substrate along the reactor chamber towards the left side, where both the unreacted precursor and reaction by-products are evacuated. The overall appearance also suggests a certain axial (lengthways) symmetry due to a quite good reactor fluid dynamic, although different samples show a slight asymmetric behaviour probably due to local deviations from the flatness of the stainless steel mesh, thus generating local turbulences.

Under our deposition conditions (T ≤ 400 °C), the growth rate increases exponentially with temperature, suggesting a kinetics control of the TiO_2_ deposition process, assuming an Arrhenius-type dependence on deposition temperature [15]. Moreover, the probability of homogeneous gas-phase reaction due to pre-reaction of TTIP is excluded. According to Ahn et al. [16], the reaction mechanism for the formation of TiO_2_ thin films, using TTIP as single-source precursor (no need of a further addition of an oxidant) could be identified by one of the following reactions, depending on deposition temperature (Equations (1) and (2)):

Ti(OC_3_H_7_)_4_  → TiO_2_ + 4C_3_H_6_ + 2H_2_O   for T ≥ 400 °C (1)

Ti(OC_3_H_7_)_4_ → TiO_2_ + 2C_3_H_6_ + 2HOC_3_H_7_   for T ≤ 400 °C (2)

Both Wu et al. [17] and Fictire et al. [18] also confirmed that the main organic decomposition by-products are isopropanol, propene and acetone. The proposed mechanism involves a possible transition state characterized by the presence of a six-membered ring. An oxygen of an isopropoxide ligand interacts with a terminal hydrogen of another isopropoxide group. This phenomenon occurs for either an adsorbed precursor or a partially reacted surface intermediate [17].

The deposited TiO_2_ thin films exhibit a wide variety of colours as a function of the film thickness, as already reported [19]. In this case, the interference edges can be effectively used for the qualitative evaluation of the TiO_2_ whole deposition. For the sake of simplicity, the film thickness can be evaluated taking advantage of interference edges starting from the extreme left side, where no deposition occurred (0 cm referring to the scale ruler in Figure 1b). In the range from 1 to about 2 cm, a variation from gold to dark and light blue occurs, thus indicating a 20–60 nm thick TiO_2_ film [19]. Meanwhile, from 3 to 10 cm, a rapid sequence of colours can be observed, testifying the thickness increase up to about 300 nm. The film thickness reaches a relative maximum value at about 20–22 cm (about halfway down the ruler), then a slight decrease is observed until 35–38 cm. Finally, a new and constant film thickness increase is observed when reaching the right side of the sample (corresponding to the precursor entrance into the MOCVD reactor during the deposition).

**Figure 2:**
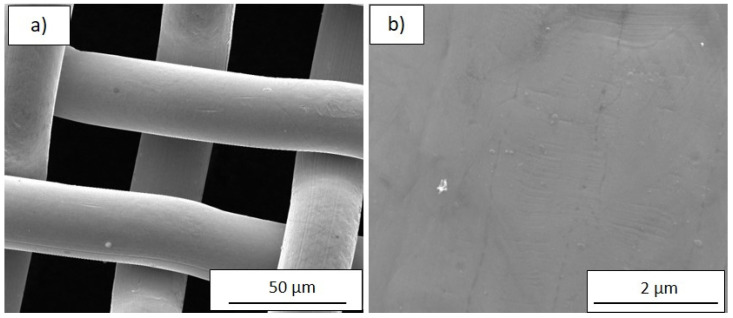
SEM images at (**a**) low (2000×) and (**b**) high (50,000×) magnification of the bare SSM substrate.

Since the simple colour-thickness correlation loses its accuracy for thicker zones, a systematic investigation by SEM of the different regions was carried out. The bare stainless steel weave was also investigated by SEM to estimate the substrate contribution to the film morphology. Figure 2a shows a large view of the bare mesh, while at higher magnification (Figure 2b) the smoothness of the surface is evidenced; only some linear and parallel grooves probably due to wire drawing process are visible.

**Figure 3:**
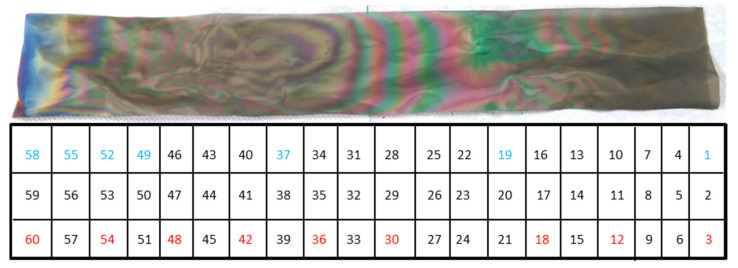
TiO_2_-SSM sample and the schematic partition enumeration. The total length of the mesh is 40 cm. The sample numbers marked in red were used for SEM and XRD analyses, while the blue ones for the photocatalytic tests.

The TiO_2_-SSM sample was prepared (40 × 6 cm^2^) and divided into enumerated small squares 2 × 2 cm^2^ as shown in Figure 3. In order to provide a significant and representative investigation of the surface, squares #3, #12, #18, #30, #36, #42, #48, #54, and #60 were analysed via SEM for the definition of the film morphology and thickness. Neither delamination nor breaks in the film were observed, thus testifying to the high quality of the deposition.

**Figure 4:**
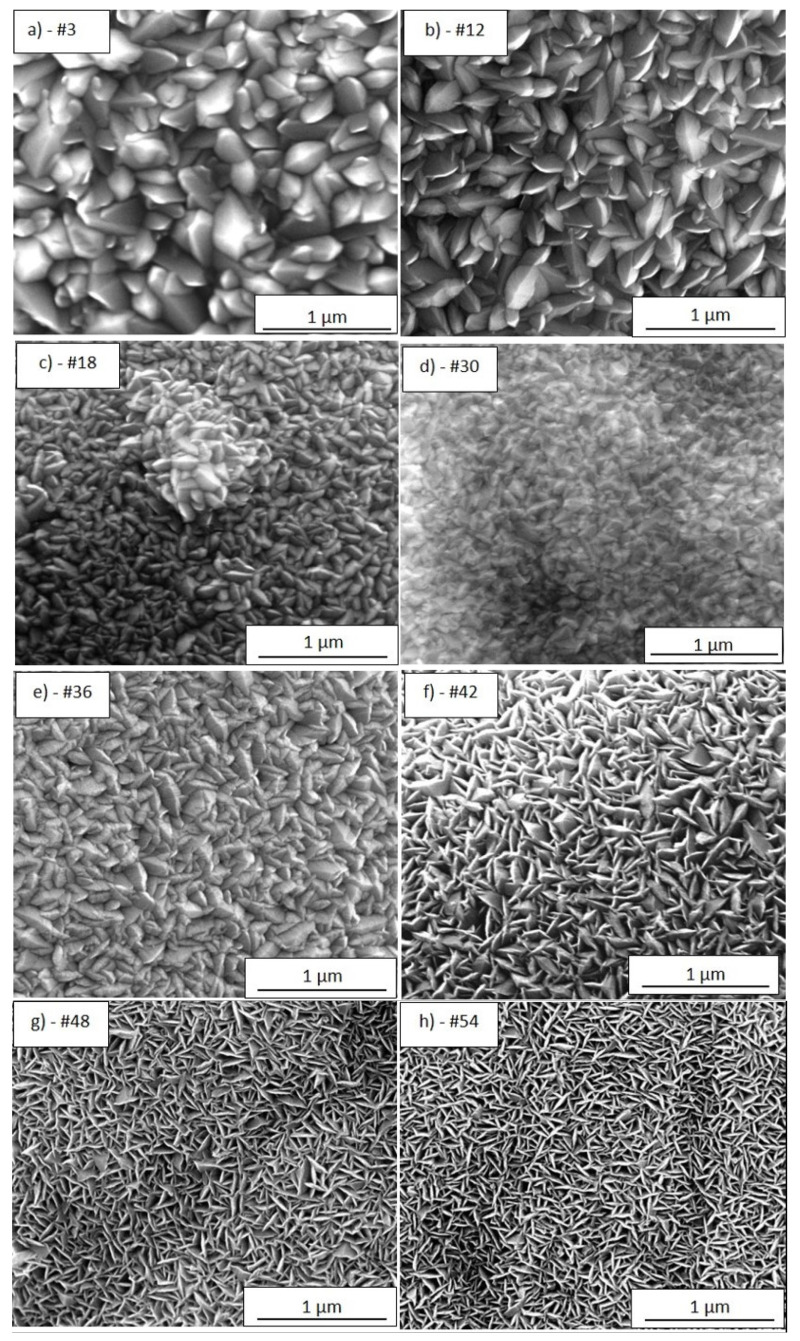

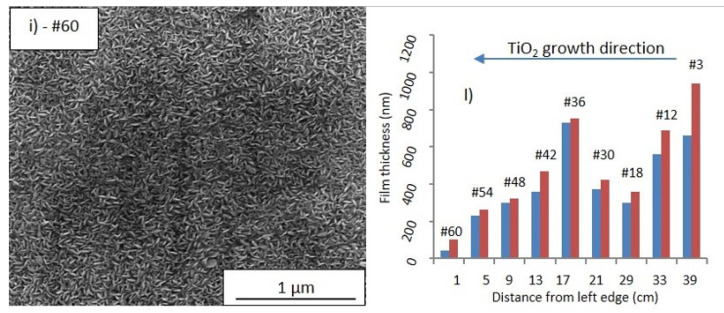
SEM images (100,000× magnification) of the TiO_2_-SSM portions #3 (**a**), #12 (**b**), #18 (**c**), #30 (**d**), #36 (**e**), #42 (**f**), #48 (**g**), #54 (**h**), and #60 (**i**). TiO_2_ film thickness (blue = minimum value, red = maximum value within the analysed portion) as a function of the position (distance from left edge, i.e., left side of the sample) for the correspondent portions (**l**).

Figure 4a–i show the typical SEM morphology for each analysed portion of the sample TiO_2_-SSM, while Table 1 resumes the main data obtained from the characterisation of the investigated portions. In general, MOCVD process allows the coating of the overall mesh (upper and lower face) while also covering the inside walls of the holes. For a better and more immediate comprehension, all of the images are taken with the same magnification. The analysis of the regions #3 and #12 (Figure 4a,b, respectively) indicates a compact structure of densely packed crystals with a prismatic faceted aspect about 300–400 nm wide (reactor temperature in such zone = 350 °C). The film thickness reaches 660–940 nm for region #3 and 560–690 nm for region #12. It is worthwhile to underline the wide thickness range and the indented surface in the portion #3 (the widest among all). Portion #3 is close to the reactor entrance; thus, some turbulences in reactants stream supply could justify the observed behaviour.

Figure 4l summarizes the minimum and maximum film thickness determined as the average value of at least three sections in different areas of the SEM images, not shown for sake of brevity. The film morphology of the portions #18 and #30 (Figure 4c,d, respectively) indicates smaller prismatic grains. In detail, the film thickness of the portion #18 reaches a relative minimum (300–360 nm), while in portion #30 the thickness newly increases (370–420 nm). This behaviour is explained by taking into account the gradual depletion of the precursor in the gas stream (thickness reduction for portions #12 and #18), as well as the effect of the rising temperature in the second third of the reactor length (T = 380 °C), which produces a boost in the reactant deposition (portion #30). The portion #36 (Figure 4e) results in a larger prismatic faceted grains and a larger film thickness (730–750 nm), thus confirming a temperature accelerated reactivity. The morphology of the portion #42 (Figure 4f) shows a different feature: a kind of petal-like structure is now evident. This morphology is well visible also in the portions #48 and #54 (Figure 4g,h, respectively), but the size of the petals tends to decrease with the portion number. The linear dark tracks evident in Figure 4g,h could be reasonably related to the substrate morphology contribution (i.e., the linear grooves observed in Figure 2b), and is indicative of high conformality of the TiO_2_ deposition. Finally, in the last portion #60 (Figure 4i) the petals appear as very small grains and the film thickness gradually decreases. The trend of the film thickness (Figure 4i) observed from portions #42 to #60 reflects the gradual depletion of the precursor content in the reactant stream (reactor temperature in such zone = 400 °C).

Furthermore, it is worthwhile to underline that the film morphology and thickness are the overall result of both reactor temperature and precursor feeding and then of a dynamic equilibrium between the precursor depauperation and the increasing substrate temperature along the growth direction. Indeed, the portions #18, #30, #42 and #48, despite having similar thicknesses, show different morphology due to the different reaction conditions.

**Figure 5:**
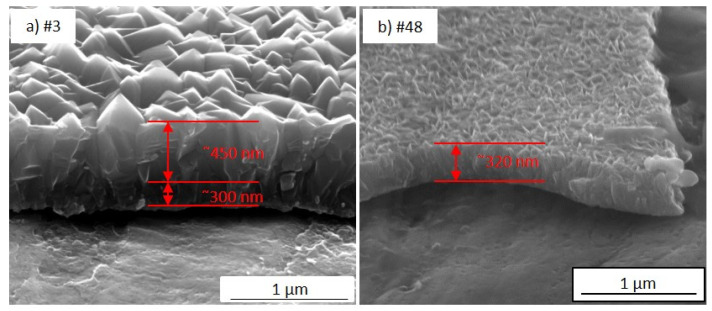
SEM images of the cross-section of the portion #3 (**a**) and #48 (**b**) (100,000× magnification).

The cross-sections of different TiO_2_-SSM portions (Figure 5) suggest a dense inter-penetration of the prismatic-like structures. The growth does not appear as vertical independent columns, but rather as faceted columns thinner at the bottom and wider on the top, similar to those observed by Jung et al. [20]. The switch between the thinner and wider columns growth appears to be at about 300–350 nm, as shown in Figure 5a for portion #3. On the other hand, no significant variations are evident in the section of portion #48 (Figure 5b).

The prismatic faceted structures seem prevalent in the thicker portions (from portion #3 to #36), while starting from portion #42 the morphology shows the petal-like structure.

Despite the widely available literature on TiO_2_ by MOCVD, an accurate comparison is not easy to carry out. Careful attention must be taken when discussing film features with reference to the literature. Many different parameters such as the substrate nature and morphology (glass, silicon, steel, etc.), the growth processes (low pressure-MOCVD, atmospheric pressure-MOCVD, aerosol and/electric field assisted-CVD, pulsed-pressure-CVD etc.), the growth temperatures and/or precursor feed were employed, making any fruitful comparison difficult [21,22,23,24,25,26,27]. Furthermore, even if SEM images of the samples prepared under specific conditions are presented in the literature, a morphologic trend as a function of thickness or sample dimensions can rarely be extrapolated. More frequently, the study of the film characteristics as a function of deposition temperature is reported [22,27,28]. The deposition temperature and the amount of supplied titanium precursor influence the growth regime, thus leading to the growth of numerous small grains, or a reduced number of larger nucleation centres. Different considerations arise from the analysis of the wide-area substrate. In detail, it can be supposed that at the entrance of the reactor (i.e., right side of the sample in Figure 1b and Figure 3, temperature = 350 °C) the precursor starts a rapid nucleation resulting in a number of relative small grains (the 300 nm-section in Figure 5a) that consolidate into larger columns during the film growth (the 450 nm-section in Figure 5a) thanks to the large availability of the precursor, thus leading to the morphology of Figure 4a,b. In the middle of the reaction chamber, the combination of temperature (380 °C) and residual amount of TiO_2_ precursor allow for a fast growth, thus obtaining a rising in film thickness with an increased number of smaller grains. Finally, in the reactor tail closeness, the higher temperature (400 °C) and the low precursor availabilityfavour the nucleation and growth of columns with almost constant width along the entire film thickness (Figure 5b). Indeed, in the final part of the sample (i.e., left side in Figure 1b and Figure 3) a high number of very small nucleation centres (the “petals”) are observed, but the remaining precursor is not enough to further increase the film thickness.

The substrate roughness (nearly smooth in this case) does not influence the deposition appearance. Siriwongrungson et al. [28] found a similar structure shape, although larger than the present work, for TiO_2_ grown on silicon nitride substrate by PP-MOCVD at 400 °C and for thinner film (200–350 nm). Arifin et al. [27] deposited TiO_2_ by MOCVD on silicon at 400 °C obtaining a thin film (about 100 nm) with uniform small circular grains. Brevet et al. [23] observed a pyramidal shape morphology for 500 nm thick TiO_2_ film on Si by MOCVD. Krumdieck et al. [9,29] deposited very thick TiO_2_ film (2 µm) on fused silica by PP-MOCVD and obtained (T = 400 °C) prismatic top structures about 200–250 nm wide. Although with different reaction conditions in terms of pressure and precursor supply (T = 350 °C and 20 min deposition time), Zhang et al. [30] deposited TiO_2_ film on 310S stainless steel by AP-MOCVD, observing a similar morphology with faceted prismatic grains.

In the present case, the system is more complex because of the wide area of the substrate. As specified in the experimental section, the reactor temperature was gradually increased from inlet to outlet in order to modulate the precursor reactivity along the chamber to compensate for the precursor depletion along the reaction chamber. Indeed, if a constant temperature is chosen, the precursor would react with the same kinetic, thus progressively depleting itself and presenting an almost linear decrease of film thickness from inlet to outlet. The lower temperature of the inlet leads to a lower reactivity, thus preserving more precursor for the reactor tail. Increasing the temperature along the chamber leads to an increased reactivity of the residual amount of precursor, thus obtaining a sufficient film growth. At the very end of the reactor, the residual precursor is forced to react via a further temperature increase. The selected set of experimental parameters is the best compromise for guaranteeing as much as possible the deposition of a high surface photoactive sample. It appears reasonable to suppose that the different reaction temperatures affect both the nucleation and the crystallite growth rates.

**Figure 6:**
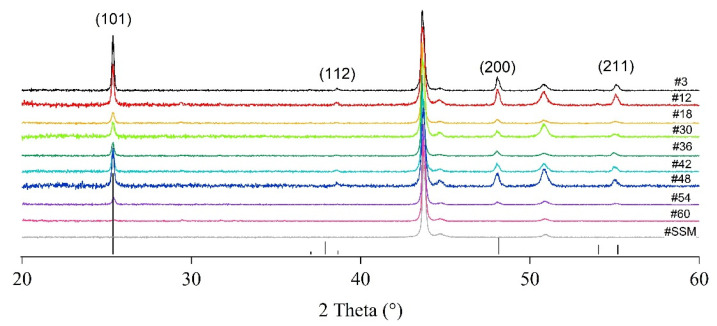
XRD patterns for the bare SSM and the different portions of the TiO_2_-SSM. The anatase TiO_2_ pattern (black vertical bar, ICDD #01-073-1764) is also reported for comparison.

The XRD patterns of the different portions of TiO_2_-deposited substrate are shown in Figure 6 as well as the XRD pattern of the bare SSM. In sample #60, TiO_2_ diffraction reflections are not evident. This behaviour is not unexpected since the film thickness is quite low, and the crystallite domains could be amorphous or under the detection limit of the experimental set-up. Besides the presence of the substrate signals (vertical dashed lines at 43.7°, 44.7° and 50.9° 2θ degrees, Figure 6), the outcomes show that TiO_2_ is polycrystalline and grows only in the anatase phase, according to the ICDD reference card # 01-073-1764, while no rutile or brookite are detected. Moreover, it is worthwhile to underline that the relative intensity of the (200) reflection plane is always slightly higher than the corresponding peak in the ICDD card. This intensity change indicates an increase in the crystallographic (200) orientation. However, in XRD patterns of incompletely oriented materials, other (hkl) reflections still occur. The degree of any possible preferred orientation (F_hkl_) may be semi-quantitatively evaluated by the Lotgering method [31] using the following Equation (3) (compiled as an example for “00l” planes):

F_00l_ = (P_00l_ − P_0_)/(1 − P_0_) (3)

where F_00l_ is the Lotgering factor or texture fraction (ranging from 0 to 1), P_00l_ = ∑I_00l_/∑I_hkl_ and P_0_ = ∑I°_00l_/∑I°_hkl_ (i.e., the ratio of the peak intensity of (00l) orientation and the sum of all peak intensities for the textured films and for the randomly-oriented specimen, respectively). According to Lotgering, the quantity F, considered as a measure of the degree of orientation, is taken as a quality factor for the orientation. As an example, the ratio of the intensities of the (00l) and (hkl) reflections increases with improving orientation. When F is equal to 0 corresponds to a random orientation of the specimen, while F equal to 1 relates to a perfect orientation.

For all the deposited layers, the Lotgering factor (F_200_) is estimated from the XRD patterns recorded at RT by considering the reflections in the 2θ range 20°–60°. The F_200_ factors calculated are listed in Table 1. The maximum F_200_ calculated value is 0.2, while the modal value is 0.1, which indicates a general poor (200) preferential orientation [31].

The application of Scherrer’s equation on the main reflections deriving from (101) and (200) planes provides the crystallite sizes as shown in Table 1. The samples #3 and #12 show the highest crystallites values due to the high precursor availability in correspondence of the entrance of the reactor (151 and 107 nm for the (101) reflection, respectively), whereas from portions #18 to #54 the crystallites sizes (70 and 81 nm for (101) and (200) reflections, respectively) appear as the result of the action of different competitive factors. Indeed, the surface diffusion of the species and the deposition kinetic are strongly dependent on the reactor temperature and the precursor feeding. Moreover, the high reactor area of deposition contributes to make harder the punctual justification of the obtained results. For portion #60 no values can be calculated because of the low intensity of the diffraction pattern.

As specified above, a detailed comparison with literature data is very hard to be carried out. However, with very similar experimental conditions, but using a soda-lime glass as the substrate, Gerbasi et al. [32] obtained pure TiO_2_ anatase but they found an opposite trend: films are poorly oriented at lower thickness, while the (200) plane became very intense for thicker ones. Jung et al. [20] deposited TiO_2_ on alumina balls by MOCVD at different temperature, finding (112) oriented anatase as main phase, although brookite was also present. In this case, a deeper comparison cannot be carried out since no correlation with the film thickness was provided.

### 3.2. SSM Substrate and TiO_2_-SSM Deposited Samples Photocatalytic Activity

In order to provide a simple, reproducible, and comparable data set concerning the photocatalytic properties of the prepared samples as a function of film characteristics, the bare stainless steel substrate (SSM) and the portions #1, #19, #37, #49, #52, #55, and #58 (with reference to Figure 3) were analysed under ISO 10678:2010 conditions. Each test was repeated three times.

This protocol is based on the evaluation of the photodegradation of MB dye, the degradation mechanism of which is still under investigation. It is well accepted that regardless of photocatalysts, different activating radiation sources (or radical primers) during the early stages of the degradation MB-related molecules such as Azure A, Azure B and Azure C (identical MB structure, save the pendant methyl groups) and several hydroxylated and oxidised products are observed [33,34,35,36,37,38,39]. On the other hand, with the progress of the process, the identification of the intermediates becomes more difficult because of the low amount of the different species, and the degradation mechanism becomes more speculative, trying to suppose the way for the rings opening and the complete mineralization.

As well known, when activated by a suitable radiation, the photocatalyst generates electron-hole pairs, which are the first actors in the photocatalytic degradation of the dye. Tao et al. [40] suggested that superoxide radicals and holes are the main reactive species in anatase-rich photocatalysts, while hydroxyl radicals have a minor role. On the other hand, Trandafilovic et al. [36] stated that in anoxia conditions, the action of the holes lead only to the MB reduction to the leuco form and hydroxyl radicals are also essential in further MB mineralization. Thus, although the mechanism of photodegradation of MB is still object of debate, the adopted protocol is universally used to evaluate the photocatalytic performance of a photocatalyst.

**Figure 7:**
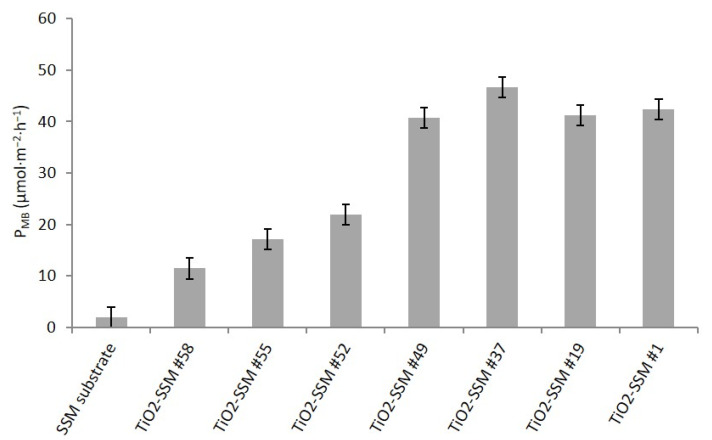
Photocatalytic activity by ISO 10678:2010 for the investigated samples as a function of the position in the large TiO_2_-SSM substrate.

Figure 7 resumes the photocatalytic activity (P_MB_) calculated according to ISO 10678:2010 for methylene blue removal from an aqueous solution. The P_MB_ of the bare SSM substrate appears negligible, as expected. The thinnest sample (#58, 40–100 nm thickness) shows a limited activity and the P_MB_ gradually increases with the TiO_2_ thickness following a non-linear trend. Indeed, although the activity of samples #58, #55 and #52 shows a progressive linear gain, sample #49 shows a significant increase. Starting from portion #49 (thickness about 250–300 nm), the P_MB_ seems to reach a stable value (over 40 µmol·m^−2^·h^−1^) and no further activity increase is observed when thicker films (portions #37, #19, and #1) are investigated. The observed data suggest that a minimum film thickness of at least 250–300 nm is required to reach the best performance of the TiO_2_-SSM overall sample. The photocatalytic activity towardsMB degradation is very interesting when compared with literature [41].

To the best of the authors’ knowledge, thin film photoactivity is not deeply discussed in the literature and it is restricted to a set of few parameters. As pointed out by Eufinger et al. [42], it would be possible to evaluate the effect of film thickness on photoactivity only if all the other parameters (such as crystallite dimensions, morphology, defectiveness, etc.) remain unchanged. This is practically very hard to pursue: commonly, when preparing a set of samples with increasing thickness, the synthesis leads to a multi-variation of the film characteristics (i.e., grain morphology and shape). Consequently, the measured photoactivity reflects the combined effect of the occurred changes and no univocal conclusion can be drawn. The only way for a proper evaluation is precisely to know the film characteristics and their variation. In the present case, the film structure is always anatase, although crystallite sizes slightly change with film thickness. Concerning the film morphology, on the other hand, the petal-like structure of the thinnest portions evolves towards faceted grains with the film thickness. Based on simplified geometrical considerations by modelling the sample surface as a series of pyramids-like structures and calculating their surfaces with the pyramid sides and heights evaluated from SEM images, the specific area of samples #54 and #48 is estimated to be almost equal to that of sample #12 (#12 has less but higher pyramids, while #54 and #48 have more but lower ones). It follows that the specific area cannot be invoked for the photoactivity increase.

As a general consideration, the photocatalytic performance is affected by several aspects such as the crystallinity of the catalyst, its particular crystallographic structure, the specific surface area, the electron-hole pairs recombination rate, and, for a thin photocatalytic film, the film thickness. Focusing on TiO_2_, it is well known [3] that the photocatalytic efficiency is favoured by a high crystallinity degree (defects are recombination centres for electron-hole pairs). The anatase phase provides better results than the rutile phase, but their heterojunction gives a further improved performance, while other crystallographic phases (for example, Brookite or TiO_2_-B) are not very interesting. In the present work, the adopted CVD procedure allows to obtain a crystalline and pure anatase phase with no rutile presence (the higher temperature required for obtaining rutile involves a quenching effect of the substrate mesh on the TiO_2_ performance). As demonstrated, the photocatalytic activity reaches a steady state when both crystallinity and film thickness reach appropriate values. So, there is no a single key factor affecting the photoactivity, but a synergistic cooperation of several parameters, and the worst of them is the critical bottleneck.

The nanostructured materials (both as film and powder) are usually characterized by an increase of the defects, which typically act as recombination centres. The optimum compromise consists in the right balance between the nano-size (i.e., gain in specific surface area and in non-commonly exposed crystallographic planes) and the detrimental increase of crystal defects [42]. In the present case, it has been observed that the film thickness increase does not lead to a significant crystallite growth. Indeed, only sample #3 shows a clear increase in crystallite size, but the photoactivity measured on its equivalent (sample #1) does not show any appreciable variation with respect to the less thick and smaller crystallites samples.

Jung et al. [43] evaluated the photocatalytic activity of a TiO_2_ film prepared by CVD with respect to methylene blue degradation, concluding that the optimal film thickness is in the 3–5 µm range. This appears very far from the results of this work which agrees with the outcomes from Tada et al. [44] and Nam et al. [45]. Both prepared TiO_2_ films by dip-coating, but Tada found an optimal thickness of about 140 nm (for 1,3,5,7-tetramethylcyclotetrasiloxane degradation), while Nam in the range 360–430 nm (for trichloroethylene degradation). The increase in the film thickness involves the increase of the absorbed incident light and then an increase in the number of photoelectron-hole pair generation. The trend is not monotonic, but a plateau is reached for a thick sample: most of the incoming light is absorbed in the first 100 nm of the film [42]. Moreover, the created photoelectron-hole pairs need to diffuse to the sample surface to activate the photo-degradation process. Anyway, when the generation depth is too high, the diffusion towards surface is less probable thus leading to an early recombination of the pairs. It should be observed that both the different film preparation techniques and the experimental setup for photocatalytic evaluation lead to different film properties and reactivity. Moreover, neither Tada nor Nam discussed the microstructure of the prepared samples, essential features for a useful comparison.

In the present case, it can be hypothesised that during the early stages of the film growth (about the first 100 nm), the generated TiO_2_ film is not adequate because of its poor crystallinity or too small crystallites with high defectiveness, thus leading to a fast recombination rate of the photoelectron-hole pairs. Even if it is not possible to calculate the crystallites size of TiO_2_ film in portion #60 from XRD measurement, evaluation from SEM images clearly shows a growing film. The increase in film thickness, reaching values between 200–300 nm, exceeds the minimum critical crystallite dimensions increasing the photocatalytic performance.

The main outcomes of the present work are related to the definition of the minimum film thickness that guarantees an adequate and constant photoactivity over a large part of the wide-area sample. The definition of the thinnest film thickness allows preparing the supported photocatalyst with the lowest load of the active compound, thus avoiding the wastefulness of such material. It is worth to underline that the employed reactor uses the simplest and cheapest possible configuration but, of course, with unavoidable drawbacks. A better film homogeneity in terms of thickness and morphology, in fact, could be obtained by using a reactor with a different geometry. As an example, a top inlet shower-like precursor entrance, possibly with multiple entrances, joint to a rotating sample holder, could guarantee a more homogeneous thickness coverage of the substrate. Nevertheless, the homogeneous heating of the sample, as well as the reactor opening-closing operations, appears more complicated and need to be opportunely designed. Therefore, the use of such improved high-performance reactors appears to not strictly be necessary since it is here demonstrated that the photocatalytic activity appears equally constant.

## 4. Conclusions

A TiO_2_ thin film was prepared by LP-MOCVD on a large area stainless steel mesh substrate. The wide sample was divided into smaller portions for multiple film characterisations. From the XRD analysis, it was demonstrated that TiO_2_ always grows as anatase. SEM investigations highlight that the thicker zones of the film (reactor entrance) grow as faceted structure (although the cross-sections suggest that the early stages of the growth could start with petal-like structure), while the thinner ones show a petal-like morphology. For both regions, the crystallites size is quite constant. The film thickness is not constant throughout the whole sample area, but it depends on the position inside the tubular reactor. Despite the slight observed variations, the photocatalytic performance is mainly determined by the film thickness, and it is stable after a critical minimum thickness of 250–300 nm is reached. Because of this, only the few centimetres at the end of the growth zone need to be excluded.

The obtained supported catalysts will be successfully exploited in a photo-reactor for water or air treatment for pollutants removal. The use of a supported photocatalyst can significantly reduce the complexity of the plant in terms of post-treatment operations, since no post-filtration or settling is necessary for the recovery of the catalyst.

## Figures and Tables

**Table 1:** Main experimental data obtained for the TiO_2_-SSM portions characterisation. Concerning the film thickness, the minimum and maximum values are given. Crystallite dimensions (nm) determined by Scherrer’s equation were calculated with respect to the main reflections of (101) and (200) planes (the uncertainty for each datum is ± 5 nm). F_200_ is the Lotgering factor calculated for the (200) XRD reflection.

Sample	Film Thickness (nm)	D_cryst_ (101)	D_cryst_ (200)	F_200_
#3	660–940	151	91	0.1
#12	560–690	107	75	0.1
#18	300–360	70	60	0.2
#30	370–420	74	57	0.2
#36	730–750	81	47	0.1
#42	360–470	83	57	0.1
#48	300–320	103	46	0.1
#54	230–260	81	57	0.1
#60	40–100	ND	ND	ND

## Data Availability

Not applicable.
